# Bioequivalence and Pharmacokinetics of Low-Dose Anagrelide 0.5 mg Capsules in Healthy Volunteers

**DOI:** 10.3390/biomedicines13081993

**Published:** 2025-08-15

**Authors:** Ahmet Inal, Zafer Sezer, Onur Pinarbasli, Burcu Bulut, Martin Reinsch, Wolfgang Martin, Mumtaz M. Mazicioglu, Selma Alime Koru

**Affiliations:** 1 Department of Pharmacology, Hakan Cetinsaya Good Clinical Practice and Research Center, Erciyes University, 38039 Kayseri, Türkiye; zsezer@erciyes.edu.tr; 2 ILKO Pharmaceuticals, 06800 Ankara, Turkey; opinarbasli@ilko.com.tr (O.P.); byilmaz@ilko.com.tr (B.B.); 3 Analytisches Zentrum Biopharm GmbH, 12681 Berlin, Germany; m.reinsch@az-biopharm.de; 4 Pharmakin Consulting Services UG, 89233 Neu-Ulm, Germany; wolfgang.martin@phacs.eu; 5 Department of Family Medicine, Hakan Cetinsaya Good Clinical Practice and Research Center, Erciyes University, 38039 Kayseri, Türkiye; mazici@erciyes.edu.tr; 6 IDEAL Biological Products and Pharmaceutical Consultancy Training Ltd. Sti., 06530 Ankara, Türkiye; selma.koru@idealcro.com

**Keywords:** anagrelide, bioequivalence, pharmacokinetics, LC-MS/MS, healthy volunteers, fasting, crossover study

## Abstract

**Objectives**: Anagrelide, an oral phosphodiesterase-3 inhibitor, is widely used to treat thrombocythemia. Evaluating the bioequivalence of low-dose formulations is essential to ensure consistent therapeutic outcomes while minimizing adverse effects, particularly cardiovascular events such as palpitations, tachycardia, and potential arrhythmias, which are known concerns with anagrelide therapy. This study aimed to compare the pharmacokinetics and bioavailability of a newly developed 0.5 mg anagrelide capsule with the reference product under fasting conditions y. **Materials and Methods**: In a randomized, open-label, two-period crossover design, 42 healthy Turkish male volunteers received a single oral dose (0.5 mg) of either the test or reference anagrelide capsule, with a seven-day washout period between treatments. Serial blood samples were collected over a 10 h post-dose period. Plasma concentrations of anagrelide were analyzed using a validated LC-MS/MS method. Key pharmacokinetic parameters (AUC_0_–t, AUC_0_–∞, Cmax, tmax, λz, t½, AUC–extrapol) were calculated and subjected to ANOVA-based bioequivalence analysis. **Results**: A total of 42 healthy male participants (mean age: 34.1 ± 8.9 years; BMI: 25.7 ± 2.9 kg/m^2^) completed the study without any protocol deviations. Pharmacokinetic analysis demonstrated that the test and reference formulations of anagrelide 0.5 mg were bioequivalent. The mean AUC_0_–t values were 4533.3 ± 2379.3 pg·h/mL for the test formulation and 4515.0 ± 2392.3 pg·h/mL for the reference (*p* > 0.05), while the mean Cmax values were 1997.1 ± 1159.2 pg/mL and 2061.3 ± 1054.0 pg/mL, respectively (*p* > 0.05). The 90% confidence intervals for the geometric mean ratios of AUC_0_–t (94.09–104.75%), Cmax (85.62–104.03%), and AUC_0_–∞ (94.50–105.10%) were all within the predefined bioequivalence range of 80–125%, with corresponding point estimates of 99.28%, 94.37%, and 99.66%, respectively. Intra-subject variability was 14.68% for AUC_0_–t and 26.98% for Cmax. No statistically significant differences were observed between the formulations for any of the primary or secondary pharmacokinetic parameters (ANOVA, *p* > 0.05). Regarding safety, 13 treatment-emergent adverse events were reported in 11 participants (26.2%), mostly moderate-intensity headaches, all of which resolved without complications. No serious adverse events occurred, confirming the tolerability of both formulations. **Conclusions**: This study demonstrates that the test and reference formulations of low-dose 0.5 mg anagrelide are bioequivalent under fasting conditions, with similar safety and tolerability profiles. The findings support the use of the test product as a safe and effective alternative.

## 1. Introduction

Anagrelide is an oral phosphodiesterase III (PDE3) inhibitor with both antithrombotic and platelet-reducing properties, rendering it a valuable therapeutic agent in the management of thrombocythemia associated with chronic myeloproliferative neoplasms, particularly essential thrombocythemia (ET) and polycythemia vera [1,2,3]. The drug’s primary mechanism of action involves the inhibition of megakaryocyte differentiation and proplatelet formation, leading to a targeted reduction in platelet count. Unlike conventional cytoreductive therapies such as hydroxyurea or busulfan, which can affect multiple hematopoietic lineages, anagrelide selectively decreases platelet production without significantly impairing leukocyte or erythrocyte counts [4,5,6,7]. This lineage specificity offers a favorable therapeutic advantage, especially in patients who are intolerant to broader-spectrum cytoreductive agents or in those requiring long-term platelet control.

After oral administration, anagrelide is rapidly absorbed from the gastrointestinal tract and undergoes extensive first-pass metabolism in the liver, predominantly via the cytochrome P450 enzyme CYP1A2 [8,9,10]. This metabolic process produces two principal metabolites: the pharmacologically active 6,7-dichloro-3-hydroxy-1,5-dihydroimidazo [2,1-b] quinazolin-2-one, widely known as 3-hydroxyanagrelide, and an inactive metabolite, 5,6-dichloro-3,4-dihydroquinazol-2-ylamine. Both the parent compound and its active metabolite reach peak plasma concentrations (Cmax) approximately 1 to 2 h post-dose. The terminal elimination half-life (t½) of the parent drug has been reported to be around 1.7 h, while the active metabolite exhibits a longer half-life of about 3.9 h in patients diagnosed with ET [9,11,12]. These pharmacokinetic features allow for relatively rapid onset of action and predictable systemic exposure following dosing.

According to the Biopharmaceutics Classification System (BCS), anagrelide is classified as a Class II drug, characterized by low solubility and high permeability. This classification suggests that its absorption is dissolution-rate-limited, emphasizing the importance of formulation strategies for optimizing bioavailability. Anagrelide demonstrates linear pharmacokinetics within the therapeutic dose range of 0.5 to 2 mg, enabling dose-proportional increases in pharmacokinetic parameters such as area under the curve (AUC) and Cmax [13,14]. This linearity facilitates straightforward dose adjustment and supports the clinical utility of low-dose formulations. However, the safety profile of anagrelide warrants careful consideration, particularly due to its cardiovascular effects stemming from PDE3 inhibition in cardiac tissues. Commonly reported adverse effects include palpitations, tachycardia, headaches, dizziness, and gastrointestinal symptoms such as nausea, diarrhea, or abdominal discomfort. Among these, tachyarrhythmias are especially noteworthy, as they may limit tolerability in certain patients [12,15,16,17,18]. Nonetheless, clinical experience has shown that many of these adverse events tend to diminish in frequency and intensity with continued administration, suggesting the development of pharmacodynamic tolerance over time.

The aim of this study was to evaluate the pharmacokinetic characteristics and relative bioavailability of a newly developed low-dose (0.5 mg) anagrelide capsule formulation in comparison with the reference product under fasting conditions in healthy male volunteers. A capsule dosage form was selected over a conventional tablet to ensure uniform content distribution, protect the moisture- and light-sensitive active ingredient, and avoid the potential impact of compression forces on the drug’s physicochemical stability. Additionally, capsules offer easier swallowing for some patients and greater flexibility in dose adjustments, which is advantageous in early clinical development. This lower dose is clinically relevant as it may improve tolerability, reduce the incidence of dose-dependent cardiovascular side effects, and be particularly suitable for first-in-human use or for patients requiring a gradual dose escalation. This investigation is designed to determine whether the test formulation meets the regulatory criteria for bioequivalence, and assessing the pharmacokinetic profile at this low dose is crucial for ensuring safety in healthy individuals and for supporting future clinical development.

## 2. Materials and Methods

### 2.1. Ethics Approval

This study was approved by the Ethics Committee for Bioavailability–Bioequivalence Trials of Erciyes University on 6 February 2019 (Approval No: 2019/21). Final authorization was granted by the Turkish Medicines and Medical Devices Agency (TİTCK) on 18 March 2019 (Reference No: 66175679-514.06.01-E.45591). Clinical trial registration was not applicable, as per the Turkish Medicines and Medical Devices Agency (TİTCK) and ICH-GCP guidelines, because this was a bioequivalence study conducted in healthy volunteers without therapeutic intervention.

The clinical phase of this study adhered to the principles outlined in the Declaration of Helsinki (Fortaleza, Brazil, 2013), the Turkish Pharmaceutical Law No. 1262, the ICH Harmonized Tripartite Guideline for Good Clinical Practice (ICH-GCP), and the Turkish Good Clinical Practice Guideline dated 13 November 2015 [5,6,7,8]. Written and verbal informed consent were obtained from all participants prior to enrollment.

### 2.2. Investigational Products

The test product (ANAGRELIDE 0.5 mg capsule, batch no.: 1910819001) was manufactured by İlko İlaç Sanayi ve Ticaret A.Ş. in compliance with Good Manufacturing Practices (GMPs). The reference product (AGRYLIN^®^ 0.5 mg capsule, batch no.: AF9326E) was purchased from the U.S. market and manufactured by Shire US Inc. Details of both products are presented in Table 1.

### 2.3. Study Design

This was a single-center, open-label, randomized, two-period crossover study involving 42 healthy male subjects. Screening was conducted between 1 and 2 July 2019, during which 47 male Caucasian volunteers were evaluated. Forty-two subjects meeting the inclusion criteria were enrolled and completed both study periods.

Participants were admitted to the clinical unit the evening prior to drug administration and fasted for at least 10 h. On Day 1, a single 0.5 mg capsule (test or reference) was administered with 240 mL of water under fasting conditions. A standardized meal (1200 kcal) was served 4 h post-dose. Water intake was restricted to 1.5 L on the dosing day, and fluid consumption was prohibited during the 1 h pre- and post-dosing periods to minimize potential variability in drug absorption and gastric emptying time, thereby ensuring pharmacokinetic consistency.

A seven-day washout period was maintained between the two treatment periods. Subjects remained in the clinic for at least 10 h post-dose for serial blood sampling.

**Figure 1:**
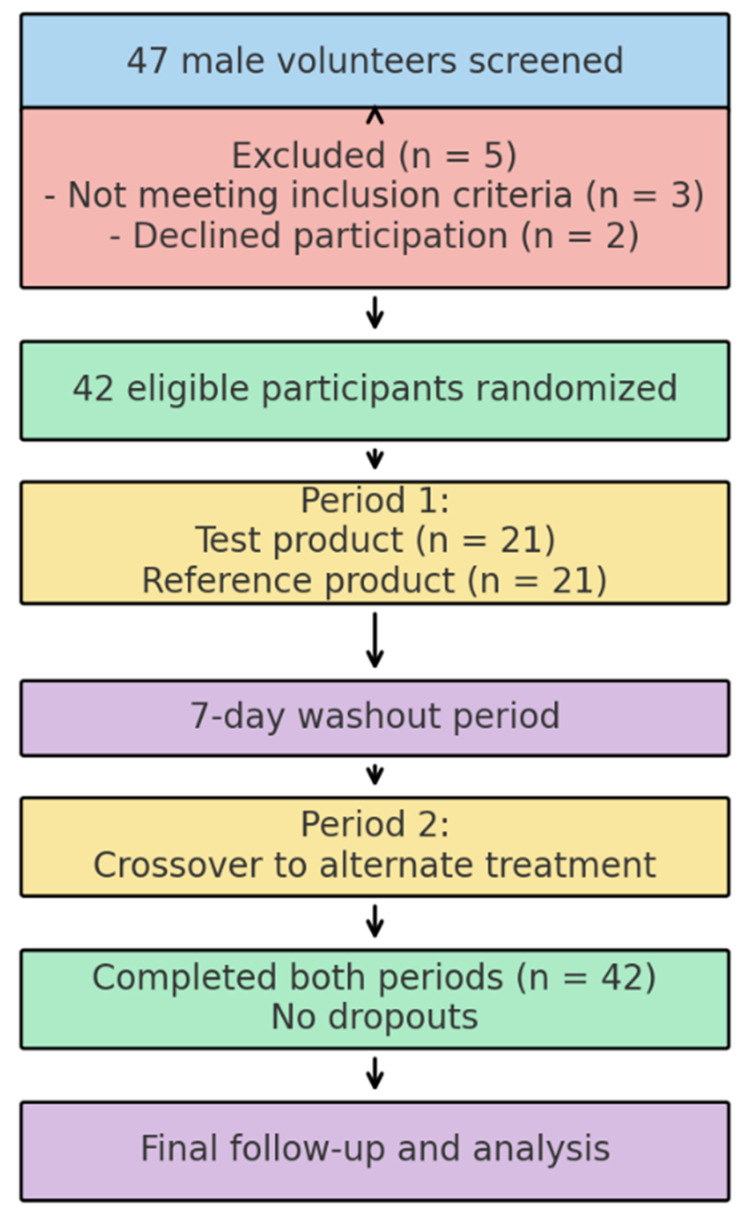
Flowchart of the study.

The inclusion criteria were healthy males aged 18–54 years with a body mass index (BMI) between 19.6 and 29.9 kg/m^2^. The exclusion criteria included smoking more than five cigarettes per day, alcohol or caffeine consumption within two days of dosing, recent use of prescription or over-the-counter medications (including herbal products), and participation in another investigational drug study within the past two months (Figure 1).

### 2.4. Blood Sampling and Sample Handling

Blood samples (~7 mL) were collected via indwelling catheter or venipuncture at the following time points: pre-dose (0 h) and 0.25 h, 0.5 h, 0.75 h, 1 h, 1.25 h, 1.5 h, 1.75 h, 2 h, 2.33 h, 2.67 h, 3 h, 3.5 h, 4 h, 5 h, 6 h, 8 h, and 10 h post-dose. Samples were collected in K2EDTA tubes, stored at 4 ± 2 °C for up to 20 min, and centrifuged at 3000 rpm at 4–7 °C for 10 min. Plasma was transferred into labeled polypropylene tubes (≥1.5 mL), sealed, and frozen at below −70 °C until analysis.

### 2.5. Bioanalytical Procedures

Plasma concentrations of anagrelide were determined at Analytisches Zentrum Biopharm GmbH (Berlin, Germany) using a validated LC–MS/MS method with positive electrospray ionization (ESI^+^). The method was validated in July 2019 in accordance with EMA and FDA guidelines on bioanalytical method validation [19,20]. Validation demonstrated the method’s selectivity, intra- and inter-assay precision and accuracy, LLOQ (40.0 pg/mL), dilution integrity, carry-over, recovery, matrix effect, linearity of the standard curve (40.0–5000.0 pg/mL), and stability under bench-top, freeze–thaw, auto-sampler, extract, and long-term storage conditions. The inter-assay accuracy (bias%) and precision (CV%) for high (HQC), medium (MQC), and low (LQC) concentrations were within the acceptable ±15% range (±20% for LLOQ). Incurred sample reanalysis (ISR) met acceptance criteria in 87.5% of tested samples.

For sample analysis, anagrelide and the internal standard (anagrelide-^13^C_3_) were separated under gradient conditions with 0.1% formic acid in water and methanol using an Ascentis^®^ C18 HPLC column (Supelco, Bellefonte, PA, USA). The multiple reaction monitoring (MRM) mode was applied, monitoring the ion transitions 256.062 → 198.900 for anagrelide and 259.099 → 199.900 for the internal standard. Chromatographic data were acquired using Analyst software (version 1.5.2), and the calibration function and sample concentrations were calculated using validated dbLabCal software (version V3) with a weighted (1/x^2^) regression model.

### 2.6. Statistical Analysis

Pharmacokinetic and statistical analyses were conducted using Phoenix WinNonlin (Version 8.1.0.3530). The primary endpoints were AUC_0_–t and Cmax; secondary parameters included AUC_0_–∞, tmax, λz, t½(λz), and %AUC extrapolated. Log-transformed values of AUC and Cmax were analyzed via ANOVA (two one-sided *t*-tests) to assess bioequivalence. Bioequivalence was concluded if the 90% confidence intervals for the test/reference ratios fell within the accepted range of 80.00% to 125.00%. Non-parametric Wilcoxon tests were also used for tmax comparisons. Sample size estimation was based on the method by Diletti et al., assuming 20–25% intra-subject variability, a test/reference ratio of 0.95–1.05, and 80% statistical power. A minimum of 36 subjects was required; all 42 participants completed the study and were included in the analysis.

## 3. Results

### 3.1. Pharmacokinetic Results

All 42 participants (mean age: 34.1 ± 8.9 years; BMI: 25.7 ± 2.9 kg/m^2^) completed the study per the protocol, without protocol deviations. The demographic characteristics of the study population are summarized in Table 2.

The pharmacokinetic comparison between the test (anagrelide 0.5 mg) and reference (AGRYLIN^®^ 0.5 mg) formulations demonstrated highly similar systemic exposure in healthy male volunteers. The mean AUC_0_–t values were 4533.3 ± 2379.3 pg·h/mL for the test and 4515.0 ± 2392.3 pg·h/mL for the reference formulation, while the corresponding AUC_0_–∞ values were 4666.5 ± 2394.3 pg·h/mL and 4638.0 ± 2447.6 pg·h/mL, respectively, indicating comparable total drug exposure. The mean Cmax was slightly lower in the test group (1997.1 ± 1159.2 pg/mL) compared to the reference group (2061.3 ± 1054.0 pg/mL), though not statistically significant. Both formulations reached peak plasma concentrations at approximately the same time (median tmax: 0.94 h), demonstrating rapid absorption. The elimination rate constants (λz) and terminal half-lives (t½) were also comparable between groups (λz: 0.5222 ± 0.1338 vs. 0.5528 ± 0.1205 1/h; t½: 1.47 ± 0.75 vs. 1.32 ± 0.31 h for test and reference, respectively). Additionally, the percentage of extrapolated AUC remained within acceptable limits for both formulations (%AUCextrap: 3.29 ± 2.33% vs. 2.94 ± 1.32%). The higher standard deviation observed for the test Cmax value reflects inter-individual variability in absorption and metabolism, a known characteristic of anagrelide pharmacokinetics. Nonetheless, intra-subject variability remained acceptable (26.98%), and bioequivalence criteria were fully met (Table 3).

**Figure 2:**
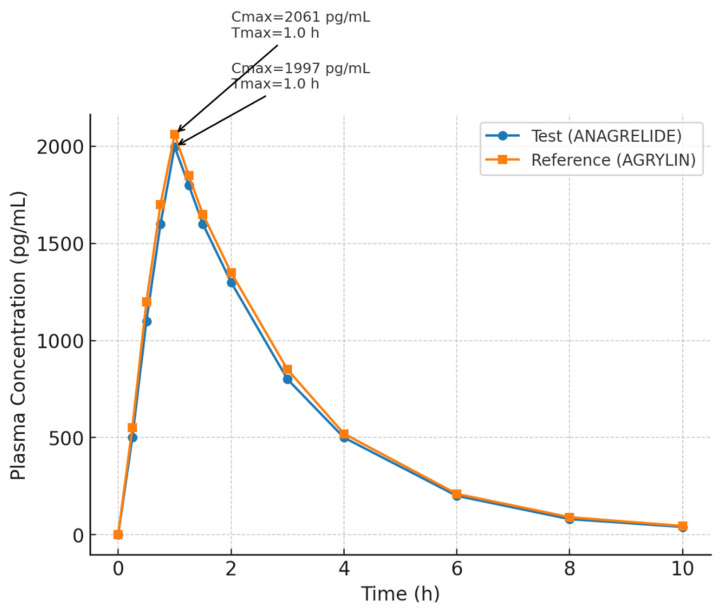
Linear plot of mean plasma concentrations (± SEM) of anagrelide following administration of test (ANAGRELIDE) and reference (AGRYLIN) formulations under fasting conditions in healthy male volunteers (*n* = 42). Cmax and tmax points are indicated on the curves.

Figure 2 illustrates the linear plasma concentration–time profiles of anagrelide following the administration of the test and reference formulations under fasting conditions. Both formulations showed a rapid rise in plasma concentrations, reaching peak levels within approximately one hour post-dose. The mean Cmax values were slightly higher for the reference product (AGRYLIN^®^; Batch No: AF9326E) compared to the test product (ANAGRELIDE; Batch No: 1910819001), although the overall profiles closely overlapped. After reaching Cmax, plasma concentrations declined in a monophasic pattern, and the elimination phases appeared parallel across both products. The standard error bars remained narrow throughout the time points, indicating low inter-individual variability (Figure 2).

**Figure 3:**
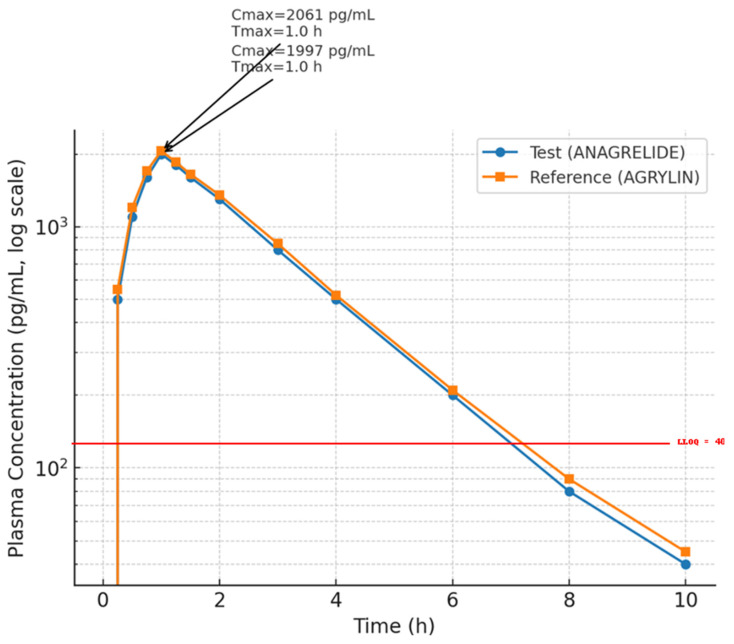
Log-linear plot of mean plasma concentrations (±SEM) of anagrelide following administration of test (ANAGRELIDE) and reference (AGRYLIN) formulations under fasting conditions in healthy male volunteers (*n* = 42). Cmax and tmax points are indicated. Note: Plasma concentrations below the lower limit of quantification (LLOQ = 40.0 pg/mL) were recorded as ‘< LLOQ’ and imputed as zero for graphical representation; values below LLOQ should not be interpreted as exact quantitations.

Figure 3 presents the mean plasma concentration–time curves of anagrelide on a semi-logarithmic scale following administration of the test and reference formulations. Both formulations exhibited a sharp increase in plasma concentrations shortly after dosing, followed by a mono-exponential decline over time. The log-linear plot allowed for clearer visualization of the elimination phase, where the slopes of the curves were nearly parallel, indicating similar elimination rates. The concentration profiles of the test (ANAGRELIDE; Batch No: 1910819001) and reference (AGRYLIN^®^; Batch No: AF9326E) products remained closely aligned throughout the 10 h post-dose period (Figure 3).

A comparison of the pharmacokinetic parameters between the test and reference anagrelide formulations is shown in Table 4. The 95% confidence intervals for the geometric mean ratios of AUC_0_–t (94.09–104.75%), Cmax (85.62–104.03%), and AUC_0_–∞ (94.50–105.10%) all fell within the predefined bioequivalence acceptance range of 80.00% to 125.00%, as specified in the FDA guidance for industry on bioavailability and bioequivalence studies for orally administered drug products [21]. The corresponding point estimates for these parameters were 99.28%, 94.37%, and 99.66%, respectively. The intra-subject variability was 14.68% for AUC_0_–t, 26.98% for Cmax, and 14.55% for AUC_0_–∞, indicating acceptable consistency in drug exposure across subjects (Table 4).

### 3.2. Safety Results

A total of 13 treatment-emergent adverse events (TEAEs) were reported by 11 out of 42 participants (26.2%) during the study. The most frequently observed adverse event was headache (*n* = 10), followed by nausea (*n* = 1), vomiting (*n* = 1), and hypotension (*n* = 1). Among the headache cases, three occurred after administration of the test product and seven after the reference product. The events of nausea and vomiting were both reported following the reference product, while a single case of hypotension occurred after administration of the test product. All adverse events were mild to moderate in severity, resolved without complications, and none were classified as serious. All events were deemed possibly related to the study medication, with the exception of hypotension, which was considered unlikely to be drug-related (Table 5).

## 4. Discussion

This study evaluated the pharmacokinetic profiles and bioequivalence of a newly developed low-dose anagrelide 0.5 mg capsule formulation in comparison with the reference product (AGRYLIN^®^) under fasting conditions in healthy male volunteers. The findings demonstrated that both formulations exhibited similar systemic exposure, as evidenced by comparable AUC and Cmax values, and met the regulatory criteria for bioequivalence. The 90% confidence intervals for the geometric mean ratios of AUC_0_–t, AUC_0_–∞, and Cmax were all within the accepted bioequivalence range of 80–125%. Additionally, both products showed a rapid absorption profile with a median tmax of 0.94 h and similar elimination characteristics. These results indicate that the test formulation can be considered therapeutically equivalent to the reference product in terms of rate and extent of absorption.

Several previous studies had investigated the pharmacokinetic characteristics and tolerability of anagrelide, particularly in healthy volunteers or patients with essential thrombocythemia. Hashimoto et al. reported that both Cmax and AUC values of anagrelide increased in a dose-proportional manner within the 0.5–2 mg range, and that the drug was rapidly absorbed, reaching peak plasma levels within 1–2 h post-administration [5]. In our study, the median tmax was 0.94 h for both the test and reference formulations, indicating rapid systemic absorption. Moreover, the observed mean terminal half-lives (1.47 h and 1.32 h) aligned with previously reported values of 1.3–1.7 h for the parent compound, further validating the pharmacokinetic reliability of the new formulation [5,22].

In their study, Petrides et al. examined the effects of caffeine and food intake on anagrelide pharmacokinetics and demonstrated that food delayed tmax and slightly reduced Cmax without altering the extent of absorption (AUC) [23]. This supported the decision to conduct the present trial under fasting conditions to eliminate confounding dietary influences and to improve sensitivity in detecting formulation differences. As in the studies by Petrides et al. and Wagstaff and Keating, our data confirmed that both the parent drug and its major active metabolite, 3-hydroxyanagrelide, followed predictable elimination kinetics without evidence of accumulation [22,24].

Regarding safety and tolerability, our results were also in line with previous investigations. Martinez-Selles et al. reported that mild to moderate headaches were the most common adverse events in healthy subjects receiving low-dose anagrelide and that no serious drug-related effects occurred [25]. This closely mirrored our findings, in which headache was the most frequently reported adverse event, accounting for 10 out of 13 total events. No participant discontinued due to adverse events, and all reactions resolved spontaneously, demonstrating support for the tolerability of the test formulation, in line with prior safety data reported for anagrelide in similar populations.

Importantly, this study provided new data on the pharmacokinetics and bioequivalence of a low-dose (0.5 mg) anagrelide capsule, a dose that had received limited attention in prior pharmacokinetic investigations. Some previously published studies, such as those by Petrides et al. and Martínez-Sellés et al., focused on higher single or multiple doses (1–2 mg), sometimes in combination with food or in patient populations with essential thrombocythemia [24,26]. In contrast, our study investigated a single low-dose formulation in a strictly controlled fasting environment, which is particularly relevant for early-phase clinical research involving this specific low-dose formulation, as it allows for assessment of safety in healthy volunteers before broader clinical use, while acknowledging that anagrelide itself is an established therapeutic agent. Furthermore, unlike earlier studies that primarily explored therapeutic effects and tolerability, our study fulfilled the criteria for regulatory bioequivalence testing using a robust analytical and statistical framework. These distinctions make this research a meaningful addition to the literature on anagrelide and support the potential interchangeability of the test formulation with the branded product.

Our fasting, single-dose (0.5 mg) crossover design and the resulting PK parameters are consistent with the AGRYLIN^®^ label. Specifically, the RLD reports dose linearity within 0.5–2.0 mg and a fasting t½ ~1.3 h at 0.5 mg; our mean terminal half-lives (test 1.47 h; reference 1.32 h) and rapid absorption (median t_max 0.94 h) closely match these values. Conducting the trial under fasting conditions aligns with the label’s description that food lowers Cmax and increases AUC. Additionally, our discussion of cardiovascular precautions is in line with label warnings, and metabolism via CYP1A2 is consistent with the label’s pharmacology. Overall, these concordances support the appropriateness of our design and the bioequivalence conclusion [27].

### Limitations of the Study

First, the population consisted exclusively of healthy male volunteers aged 18–54 years, which may limit the generalizability of the findings to female patients, elderly individuals, or those with comorbid conditions. This approach, while consistent with regulatory allowances for early-phase bioequivalence studies, was chosen to minimize potential reproductive risks in women of childbearing potential and to reduce inter-subject variability related to hormonal fluctuations, thereby increasing statistical power. Second, the study was limited to single-dose administration, and did not assess steady-state pharmacokinetics or long-term tolerability. Although single-dose designs are standard for bioequivalence studies, chronic use scenarios may exhibit different pharmacodynamic or safety profiles. Adverse events were recorded descriptively without grading according to standardized systems such as the Common Terminology Criteria for Adverse Events (CTCAE), which may limit comparability with other studies. Finally, while the study focused on the parent drug, it did not include analysis of the active metabolite (3-hydroxyanagrelide). According to the ICH M13A guidance for bioequivalence of immediate-release oral formulations, pharmacokinetic assessment based on the parent drug alone is acceptable unless the metabolite contributes significantly to pharmacological activity [28]. Nonetheless, future studies could explore metabolite kinetics to further support therapeutic equivalence. Although the present study focused on in vivo pharmacokinetic evaluation, complementary in vitro dissolution studies—particularly those assessing f_1_ (difference factor) and f_2_ (similarity factor) across multiple pH conditions (e.g., 1.2, 4.5, 6.8, and 7.4)—could further characterize the release profile and performance of the test formulation compared to the reference, and potentially enable in vitro–in vivo correlation (IVIVC) (Supplementary File, Figures S1–S3). Future research should include such dissolution testing, along with multiple-dose studies, to assess steady-state pharmacokinetics and long-term safety, as well as trials involving female participants and more diverse populations to improve the generalizability of findings.

One of the main strengths of this study was its rigorous crossover design, which minimized inter-individual variability and allowed for direct comparison between the test and reference formulations. The inclusion of 42 healthy male volunteers ensured an adequate sample size based on regulatory power calculations, exceeding the minimum requirement to detect bioequivalence with sufficient statistical confidence. All participants completed both periods of the study without major protocol deviations, contributing to the robustness and reliability of the dataset. Additionally, plasma anagrelide concentrations were measured using a validated LC-MS/MS method with a lower limit of quantification (LLOQ) of 40.0 pg/mL, ensuring precise detection even at low concentration ranges. This high sensitivity contributes to the reliability and robustness of the pharmacokinetic dataset. Another strength was the conducting of the trial under controlled fasting conditions, eliminating the potential impact of food on drug absorption and enabling a clearer interpretation of bioequivalence outcomes. This study also maintained strict compliance with ethical and good clinical practice standards, which further strengthened its methodological quality.

## 5. Conclusions

In conclusion, this study demonstrated the bioequivalence of a newly developed low-dose (0.5 mg) anagrelide capsule compared to the reference product AGRYLIN^®^ under fasting conditions in healthy male volunteers. The pharmacokinetic parameters, including AUC_0_–t, AUC_0_–∞, and Cmax, were statistically comparable between the two formulations, with 90% confidence intervals falling within the accepted regulatory range of 80.00% to 125.00%. Both formulations were rapidly absorbed, with similar tmax values, and exhibited predictable elimination kinetics. In addition, both products were well tolerated, with no serious adverse events reported.

The findings of this study supported the use of the test formulation as a therapeutically equivalent alternative to the reference product. By focusing on a lower dose in a fasting state, this research contributed important data to the existing literature, particularly in the context of dose-sparing strategies and first-in-human exposure safety. These results may also provide a foundation for future clinical trials evaluating repeated dosing or use in patient populations.

## Figures and Tables

**Table 1:** Study drugs.

Test Product	ANAGRELIDE 0.5 mg Capsule, Batch no.: 1910819001 İlko İlaç Sanayi ve Ticaret A.Ş Sancaktepe—İstanbul, Türkiye Expiry date: January 2021
Reference Product	Reference Product AGRYLIN^®^ (Anagrelide HCl) Capsule Batch no: AF9326E, Shire US Inc., Lexington, MA, USA Expiry date: July 2019

**Table 2:** Demographic characteristics of study participants (*n* = 42).

Parameters	Mean ± SD	CV (%)	Minimum	Maximum
Age (years)	34.12 ± 8.95	26.23	18.0	54.0
Weight (kg)	78.67 ± 9.95	12.65	58.0	100.0
Height (cm)	174.90 ± 5.41	3.10	164.0	186.0
BMI (kg/m^2^)	25.71 ± 2.94	11.44	19.6	29.9

**Table 3:** Pharmacokinetic parameters of anagrelide (*n* = 42, mean ± SD).

Parameters	Test Product—ANAGRELIDE 0.5 mg (Batch: 1910819001)	Reference Product—AGRYLIN^®^ 0.5 mg (Batch: AF9326E)
AUC_0_–t (pg·h/mL)	4533.3 ± 2379.3	4515.0 ± 2392.3
AUC_0_–∞ (pg·h/mL)	4666.5 ± 2394.3	4638.0 ± 2447.6
Cmax (pg/mL)	1997.1 ± 1159.2	2061.3 ± 1054.0
tmax (h)	0.94 ± 0.53	0.94 ± 0.59
λz (1/h)	0.5222 ± 0.1338	0.5528 ± 0.1205
t½ (λz) (h)	1.47 ± 0.75	1.32 ± 0.31
%AUCextrap (%)	3.29 ± 2.33	2.94 ± 1.32

**Table 4:** Comparison of pharmacokinetic parameters between test and reference anagrelide formulations (*n* = 42).

Parameter	95% Confidence Interval	Point Estimate (Test/Reference, %)	Intra-Subject Variability (%)
AUC_0_–t	94.09–104.75	99.28	14.68
Cmax	85.62–104.03	94.37	26.98
AUC_0_–∞	94.50–105.10	99.66	14.55

**Table 5:** Summary of treatment-emergent adverse events.

Drug Relationship	Adverse Event (WHO Term)	Test Product (*n*)	Reference Product (*n*)
Possible	Vomiting	0	1
Possible	Nausea	0	1
Possible	Headache	3	7
Unlikely	Hypotension	1	0

## Data Availability

The data supporting the findings of this study are available from the corresponding author upon reasonable request for up to five years after publication.

## Supplementary Materials

The following supporting information can be downloaded at: https://www.mdpi.com/article/10.3390/biomedicines13081993/s1, Figure S1: Comparative dissolution rate profile in 0.1 N HCl medium for Anagrelide 0.5 mg Capsule (Lot No: 1910819001) from the bioequivalence batch and the reference product Agrylin® (Anagrelide Hydrochloride) 0.5 mg Capsule (Lot No: AF9326E, Shire US Inc.), Figure S2: Comparative dissolution rate profile in pH 4.5 acetate buffer medium for Anagrelide 0.5 mg Capsule (Lot No: 1910819001) from the bioequivalence batch and the reference product Agrylin® (Anagrelide Hydrochloride) 0.5 mg Capsule (Lot No: AF9326E, Shire US Inc.), Figure S3: Dissolution rate profile in pH 6.8 phosphate buffer medium for Anagrelide 0.5 mg Capsule (Lot No: 1910819001) from the bioequivalence batch and the reference product Agrylin® (Anagrelide Hydrochloride) 0.5 mg Capsule (Lot No: AF9326E, Shire US Inc.).

## Abbreviations

AUC	Area under the concentration–time curve of plasma
Cmax	Maximum plasma concentration
tmax	Time to achieve Cmax
t½	Half-life
CI	Confidence interval
CRF	Case report form
EMA	European Medicines Agency
FDA	U.S. Food and Drug Administration
HPLC	High-performance Liquid Chromatography
LLOQ	Lower limit of quantification
QC	Quality control
HQC	High-quality control
MQC	Medium-quality control
LMQC	Low–medium-quality control
LQC	Low-quality control
LC-MS/MS	Liquid Chromatography–Mass Spectrometry
TİTCK	Türkiye İlaç ve Tıbbi Cihaz Kurumu
