# Supplementary material for: Bioequivalence and Pharmacokinetics of Low-Dose Anagrelide 0.5 mg Capsules in Healthy Volunteers
Source: Biomedicines. 2025 Aug 15;13(8):1993. doi: 10.3390/biomedicines13081993 (PMC12383776; doi:10.3390/biomedicines13081993)
Supplement: Supplementary file 1 [file biomedicines-13-01993-s001.zip › biomedicines-3797245-supplementary.pdf]

biomedicines

MDPI

The comparative dissolution rate profile in 0.1 N HCl medium for Anagrelide 0.5 mg Capsule (Lot No: 1910819001) from the bioequivalence batch and the reference product Agrylin® (Anagrelide Hydrochloride) 0.5 mg Capsule (Lot No: AF9326E, Shire US Inc.) are presented below.

As both the test and reference products exhibited more than 85% dissolution at 15 minutes, f_1 and f_2 values were not calculated.

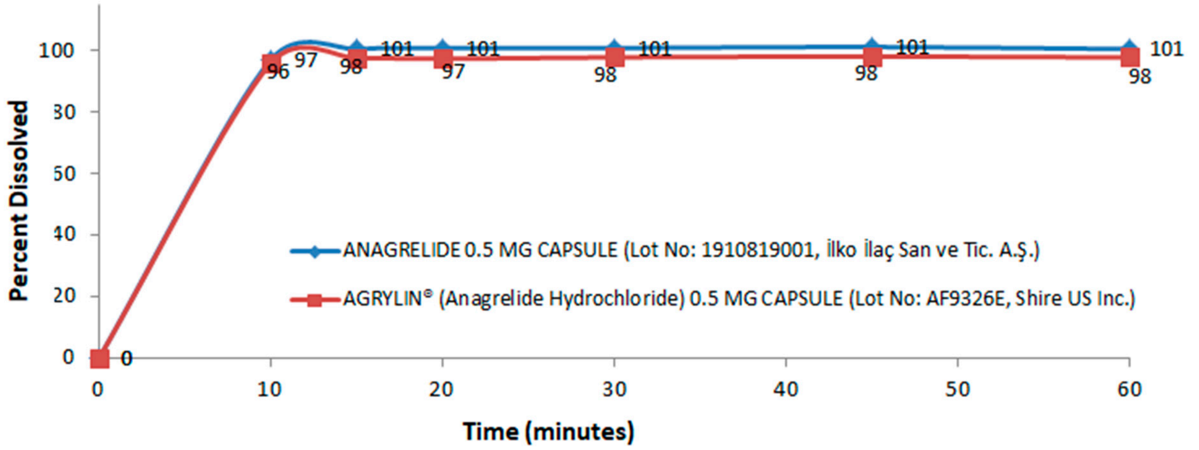

Figure S1. Comparative dissolution rate profile in 0.1 N HCl medium for Anagrelide 0.5 mg Capsule (Lot No: 1910819001) from the bioequivalence batch and the reference product Agrylin® (Anagrelide Hydrochloride) 0.5 mg Capsule (Lot No: AF9326E, Shire US Inc.)

The comparative dissolution rate profile in pH 4.5 acetate buffer medium for Anagrelide 0.5 mg Capsule (Lot No: 1910819001) from the bioequivalence batch and the reference product Agrylin® (Anagrelide Hydrochloride) 0.5 mg Capsule (Lot No: AF9326E, Shire US Inc.) are presented below.

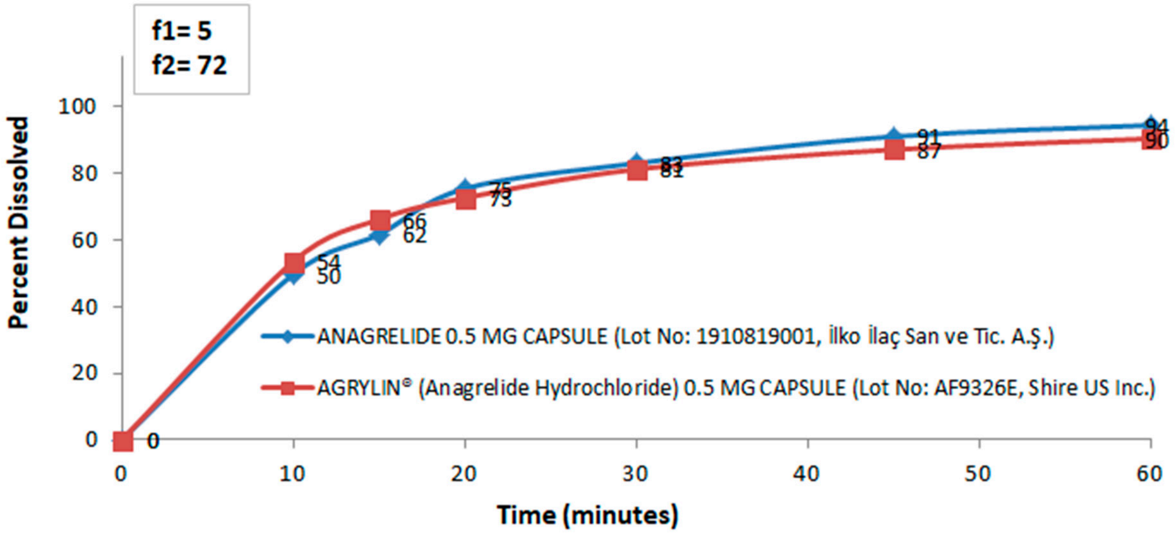

Figure S2. Comparative dissolution rate profile in pH 4.5 acetate buffer medium for Anagrelide 0.5 mg Capsule (Lot No: 1910819001) from the bioequivalence batch and the reference product Agrylin® (Anagrelide Hydrochloride) 0.5 mg Capsule (Lot No: AF9326E, Shire US Inc.)

The comparative dissolution rate profile in pH 6.8 phosphate buffer medium for Anagrelide 0.5 mg Capsule (Lot No: 1910819001) from the bioequivalence batch and the reference product Agrylin® (Anagrelide Hydrochloride) 0.5 mg Capsule (Lot No: AF9326E, Shire US Inc.) are presented below.

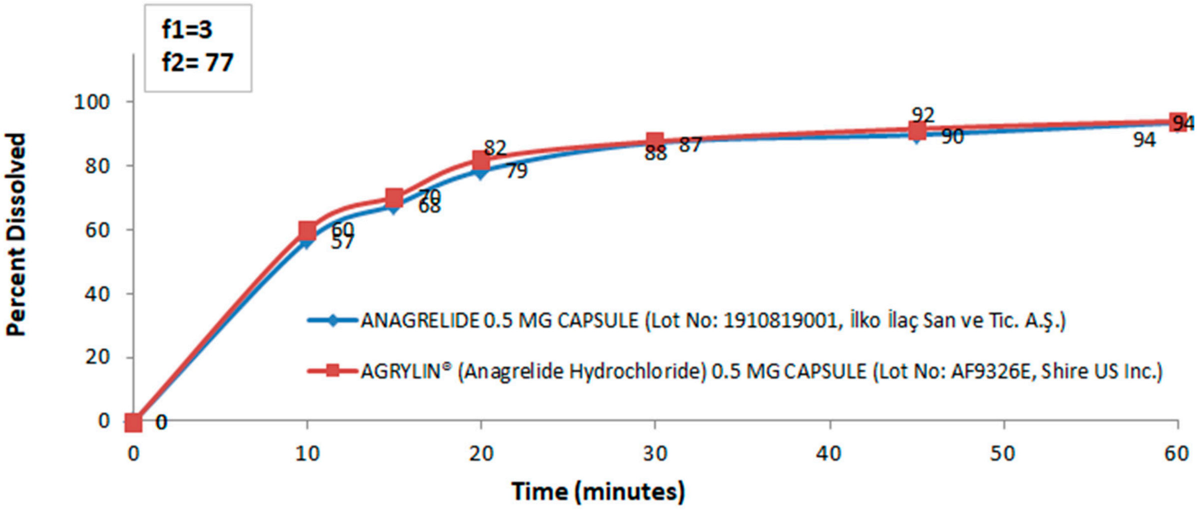

Figure S3. Dissolution rate profile in pH 6.8 phosphate buffer medium for Anagrelide 0.5 mg Capsule (Lot No: 1910819001) from the bioequivalence batch and the reference product Agrylin® (Anagrelide Hydrochloride) 0.5 mg Capsule (Lot No: AF9326E, Shire US Inc.).
